# Auditory evoked potentials in premature and full-term infants

**DOI:** 10.1590/S1808-86942011000500015

**Published:** 2015-10-22

**Authors:** Maria Angélica de Almeida Porto, Marisa Frasson de Azevedo, Daniela Gil

**Affiliations:** 1 Master's degree in human communication disorders, UNIFESP. Speech therapist of the Medical Specialty Outpatient Unit (Ambulatório Médico de Especialidades or AME) São José dos Campos; 2 Doctoral degree in human communication disorders, UNIFESP. Associate professor, UNIFESP; 3 Doctoral degree in human communication disorders, UNIFESP. Adjunct professor I, UNIFESP

**Keywords:** evoked potentials, auditory, audiometry, evoked response, hearing, infant

## Abstract

**Abstract:**

Accurate information about type, degree, and configuration of hearing loss are necessary for successful audiological early interventions. Auditory brainstem response with tone burst stimuli (TB ABR) and auditory steady-state response (ASSR) exams provide this information.

**Aim:**

To analyze the clinical applicability of TB ABR and ASSR at 2 kHz in infants, comparing responses in full-term and premature neonates.

**Material and Method:**

The study was cross-sectional, clinical and experimental. Subjects consisted of 17 premature infants and 19 full-term infants. TB ABR and ASSR exams at 2000 Hz were done during natural sleep.

**Results:**

The electrophysiological minimum response obtained with TB ABR was 32.4 dBnHL (52.4 dBSPL); the ASSR minimum was 13.8 dBHL (26.4 dBSPL). The exams required 21.1 min and 22 min, respectively. Premature and full-term infant responses showed no statistically significant differences, except for auditory steady-state response duration.

**Conclusions:**

Both exams have clinical applicability at 2 kHz in infants, with 20 min of duration, on average. In general, there are no differences between premature and full-term individuals.

## INTRODUCTION

Audiologic diagnosis within the first few weeks or months of life has become more frequent with neonatal screening programs^1^. Accurate information about the type, grade, and configuration of hearing loss is required for early audiological interventions to be successful.

Electroacoustic and electrophysiologic tests, in particular otoacoustic emissions (OAE) and the auditory brainstem response (ABR), are recommended for defining the behavioral audiogram in children aged below 6 months^2^.

The ABR with clicks is commonly used in children. Although less used in clinical settings due to increased testing time, tone burst stimuli are very useful because it is frequency specific, and therefore can be used to assess low, middle, and high frequencies^3^.

A similar advantage may be attained with the auditory steady-state response (ASSR), in which continuous, amplitude and/or frequency modulated tones evoke electrophysiologic responses that make possible a detailed and objective evaluation of hearing^4^.

Many researchers in this area have become interested in ASSR and tone burst ABR testing; these are promising tests, about which there is no consensus as to which is the best and fastest for clinical use. Existing studies have been made in adults; the Brazilian literature is particularly poor in describing these tests in term and preterm infants.

Thus, the purpose of this study was to investigate the clinical applicability of tone burst ABR and 2 kHz ASSR in infants by evaluating the time required for each test and comparing the responses of term and preterm infants.

## MATERIAL AND METHOD

A cross-sectional, clinical, and experimental study was carried out at the clinic of the Speech Therapy School. The institutional review board of the university approved this study (process no. 0713/07). Parents and caretakers authorized the children to participate and the results to be divulged by signing a free informed consent form.

The inclusion criteria were: preterm or term infants, with normal meatoscopy in both ears, bilaterally present transient evoked otoacoustic emissions, presence of the cochlear-palpebral reflex at 100 dBSPL, and click stimulus BAPE within normal limits for each child's age. Absence of one or more of these criteria excluded of the subject from this study. A Welch Allyn Pocket Junior otoscope was used for performing meatoscopy, and a Smart EP (Intelligent Hearing Systems) device was used for OAE and click stimulus BAPE. The cochlear-palpebral reflex was investigated using an agogo. Thus, middle ear conditions, poor outer ear cell function, and neural/central hearing loss were excluded.

A statistics professional defined the minimum sample number at 30 subjects. Initially, 68 infants were selected for the study from October 2007 to July 2008. Twenty-three subjects were excluded by not meeting one or more of the inclusion criteria; testing was not concluded in a single session in nine subjects, who did not return to complete the tests.

Thus, the sample comprised 36 infants that were allocated into two groups according to the gestational age. The preterm group (gestational age < 37 weeks) comprised 17 infants (12 female and 5 male). The term group (gestational age from 37 to 41 weeks) comprised 19 infants (8 female and 11 male).

The Smart EP device was also used in tone burst ABR and ASSR testing. Before testing the skin was prepared with Nuprep (an abrasive paste). ECG conductive adhesive Medi-Trace electrodes (Kendall) were placed so that recording was ipsilateral to the stimulated ear; the impedance was < 5 kΩ. The electrodes were placed as follows: active electrode on Fpz, reference electrode on M1 or M2, ground electrode on the contralateral mastoid to the stimulated ear. Subjects remained sleeping naturally on their caretaker's lap during testing.

The order and ear for starting the test were random; tone burst ABR was done first on one infant, and ASSR was done first on the next infant, on the free ear depending on how each child was positioned on the lap of the caretaker. The test parameters were those of the manual and the pertinent literature (Frame 1, Frame 2). Because the two tests are time-consuming and were done during natural sleep, we chose to record a minimum electrophysiologic response at 2 kHz from each ear separately; the most reliable and lower thresholds are found at this frequency^5^.

**Frame 1:** Tone burst ABR parameters used in this study.

Stimulus	*Tone burst - 2000 Hz, 4 ms duration (2-0-2)*
Window	*Exact Blackman Envelope*
Stimulus presentation rate	27.7/s
Polarity	Rarefied
Transducer	ER 3A In-ear phones
Intensity	80, 60, 40 e 30 dBnHL
Filters	30 - 1500 Hz
Amplification	100,000 times
Analysis time	25.6 ms
No. of stimuli	2000
Response detection	Subjective analysis with reference patterns (ex: latency/intensity function)

**Frame 2:** ASSR parameters in this study.

Carrying frequency	Tone pip at 2 kHz
Stimulus modulation	97 Hz right ear / 93 Hz left ear
Transducer	ER 3A in-ear phones
Intensity	20 dBSPL with 10 dBSPL increments to seek the minimum response level
Filters	30 - 300 Hz
Amplification	100,000 times
Sweeps	400 (responses updated every 40)
Detection of responses	F statistical technique

This choice was also based on a pilot project with 10 infants that checked test feasibility relative to the number of frequencies and intensities that were studied.

In this pilot project, tone burst ABR and ASSR were recorded at 500, 1000, 2000, and 4000 Hz, and difficulties resulting from test duration (about 3 hours) were checked. This difficulty was still found when reducing the test to the frequencies 500 and 4000 Hz; we then decided for 2000 Hz, which was considered feasible to apply in a single session in the pilot project.

The chosen intensity was 20 dBSPL in the pilot projects, as most infants presented ASSR at this intensity; furthermore, the V wave (tone burst ABR) could not be identified accurately at 20 dBHL in any of the infants. This experiment helped define our test protocol.

The test duration was measured by two methods - a microcomputer clock and a KD-1069® chronometer (Professional Quartz Timer). The former was used to measure absolute time (Δt abs) - the test duration from the beginning (after placing the electrodes) to the end (before removing the electrodes). Eventual pauses to control test conditions and/or the infant were also computed. The chronometer was used to measure relative time (Δt rel) - it was paused each time the test was stopped.

Rarefied stimuli were presented with ER 3A in-ear phones decreasingly at 80 dBnHL, 60 dBnHL, 40 dBnHL, and 30 dBnHL. The minimum electrophysiologic response at 2 kHz was measured by visual analysis of the V wave and reproducibility in two tracings.

ASSR testing was started at 20 dBSPL with 10 dBSPL increments until obtaining the minimum response, since this was a hearing sample. ASSR testing was done with a maximum presentation number of 400 sweeps. If a response was attained with a smaller number of sweeps, the test was continued for two extra 40 sweep updates to check whether the responses persisted; this procedure avoided false positive results.

Fast Fourier transformation was applied to analyze frequency domain responses. The equipment detected the responses automatically based on F statistics, which assesses the response amplitude at the modulation frequency in relation to noise amplitude at adjacent frequencies. A response was considered positive if the signal-to-noise ratio was higher than 6.13 at a p-value below 0.05.

Descriptive statistics and non-parametric tests (Wilcoxon's test and Mann-Whitney's test) were applied in the analysis. Wilcoxon's test was applied to verify differences or similarities between left and right ears; the Mann-Whitney test was used for comparing the term and preterm groups. The significance level was 0.05 (5%); values without statistical significance were marked with an asterisk (*).

## RESULTS

The study sample consisted of 55.6% females and *44.4%* males. The mean gestational age in GT subjects was 39 1/7 weeks; at the evaluation, the mean chronological age of infants was 8 2/7 weeks. The mean gestational age in the GPT was 32 6/7 weeks; the post-conception mean age was 47 3/7 weeks. The analysis of both groups showed that there were no significant differences between right and left ears; thus, both ears were considered in the subsequent

In 72 ears, the mean tone burst ABR minimum responses in nHL and SPL were 32.4 and 52.4 dB. The ASSR means in HL and SPL were 13.8 and 26.4 dB (Table 1).

**Table 1:** Descriptive measures of minimum electrophysiologic responses measure with tone burst ABR (in dBnHL and dBSPL) and ASSR (in dBHL and dBSPL).

Test	Tone burst ABR		ASSR	
	dBnHL	dBSPL	dBHL	dBSPL
Mean	32.4	52.4	13.8	26.4
Median	30	50	17	30
Standard deviation	4.3	4.3	6.0	5.9
**Q1**	30	50	7	20
**Q3**	30	50	17	30
No. of ears	72	72	72	72
Cl	1.0	1.0	1.4	1.4

Q1 - 1^st^ quartile; Q3 - 3^rd^ quartile; No. of ears - number of ears in the series; Cl - confidence interval.

Comparing GT and GPT revealed no statistically significant differences except for the absolute and relative ASSR times; in this case, the GT had lower times for carrying out the procedures (Table 2).

**Table 2:** Comparison of results among term and preterm subjects relative to the minimum tone burst ABR and ASSR responses (in dBnHL and dBSPL), V wave latency in ms (tone burst ABR), absolute and relative times (tone burst ABR and ASSR), minimum ASSR (in dBHL and dBSPL), and the number of sweeps to measure the minimum ASSR.

		Mean	Median	Standard deviation	Q1	Q3	N	CI	***p*-value**
	GT	31.6	30	3.7	30	30	38	1.2	
TB ABR (nHL)	GPT	33.2	30	4.7	30	40	34	1.6	0.101
TB ABR (SPL)	GT	51.6	50	3.7	50	50	38	1.2	0.101
	GPT	53.2	50	4.7	50	60	34	1.6	
Wave V latency	GT	10.8	11	0.9	10.2	11.3	38	0.3	0.134
	GPT	10.6	11	1.0	9.9	10.9	34	0.3	
	GT	20.9	19	5.4	17.0	23.5	19	2.4	0.644
TB Δt abs	GPT	21.4	20	5.8	17	23	17	2.8	
	GT	18.6	17	3.3	16	20	19	1.5	0.472
TB Δt rel	GPT	19.4	19	3.9	17	20	17	1.8	
ASSR (HL)	GT	13.3	17	5.9	7	17	38	1.9	0.483
	GPT	14.4	17	6.2	7	17	34	2.1	
ASSR (SPL)	GT	26.3	30	5.9	20	30	38	1.9	0.919
	GPT	26.6	30	6.0	20	30	34	2.0	
Sweeps	GT	191.1	140	126.6	80	320	38	40.2	0.536
	GPT	203.5	160	121.6	120	310	34	40.9	
Δt abs EE	GT	18.7	16	6.6	14	22	19	3.0	0.028*
	GPT	25.7	22	13.9	19	27	17	6.6	
Δt rel EE	GT	17.5	16	5.3	13.5	19.5	19	2.4	0.039*
	GPT	23.6	20	13.3	17	22	17	6.3	

GT - term group; GPT - preterm group; Q1 - 1^st^ quartile; Q3 - 3^rd^ quartile; N - number in the series; CI - confidence interval; TB Δt abs - tone burst ABR absolute time; TB Δt rel - tone burst ABR relative time; Δt abs EE -ASSR absolute time; Δt rel EE - ASSR relative time.

* *p*-value < 0.05

The absolute time was significantly higher than the relative time in test duration (Table 3), showing that pauses inevitably occur. The tone burst ABR mean times were 21.1 min (±5-5) and 19 min (±3-6). The mean duration of the ASSR was 22 min (±11.1), and the mean relative duration was 18 min (±10.3).

**Table 3:** Comparison between absolute and relative times, in minutes, of tone burst ABR and ASSR.

Time (min)	Tone burst ABR		ASSR	
	Absolute	Relative	Absolute	Relative
Mean	21.1	19.0	22.0	20.4
Median	20	18	19	18
Standard deviation	5.5	3.6	11.1	10.3
Q1	17	15.5	16.75	14.75
Q3	23.3	25	20	22
No. of subjects	36	36	36	36
CI	1.8	1.2	3.6	3.4
*p*-value	<0.001*		<0.001*	

Q1 - 1^st^ quartile; Q3 - 3^rd^ quartile; No. of subjects - number of subjects in the series; CI - confidence interval.

* statistically significant

## DISCUSSION

Before starting the discussion, it is necessary to point out that comparisons between the tone burst ABR and ASSR is difficult to perform accurately, as there are differences in stimulus calibration (dBnHL for the tone burst ABR, and dBHL for the ASSR) and the response detection method in these procedures^6^. An additional issue is that most studies of these tests in children have been done with all subjects (or part of them) under sedation^3^,^7^, ^8^, ^9^, ^10^, ^11^, ^12^, ^13^, ^14^, ^15^, ^16^, ^17^. A few studies of infants in natural sleep and adults have chosen one of the ears randomly^6^,^16^,^18^, ^19^, ^20^, ^21^ or by recording the responses in two channels^22^. These choices underline the difficulty of testing infants in natural sleep, especially when the intention is to perform tone burst ABR and ASSR testing in a single session.

Similarities in the side or ears - as noted in the present study - have also been reported in the literature^7^,^23^, ^24^, ^25^.

In the complete series (n = 72 ears) the mean minimum response was 32.4 dBnHL (52.4 dBSPL) at 2 kHz for tone burst ABR in the term and preterm groups. Studies with similar groups at the same frequency have reported lower results - in other words, better threholds^7^,^16^. However, these studies had different methods compared to our study: the minimum intensity was 10 dBnHL with two expert judges establishing the presence of the V wave,^16^ and stimuli at 2000 to 6000 were used^7^. A Brazilian study^22^ using 1500 Hz showed that 30 dBnHL may be considered a normal tone burst ABR intensity for infants and infants.. Analysis of the ASSR in our complete series showed that the mean minimum response was 13.8 dBHL (26.4 dBSPL) at 2 kHz. These data concur with other published results^11^,^20^,^23^,^26^.

Very few studies have compared thresholds measured with tone burst ABR and ASSR in the same population^6^,^15^,^17^,^19^,^27^.

The choice of two measurements of test duration was made to observe the true clinical application of these tools. Sedation for tone burst ABR and ASSR testing in infants is not a common practice in Brazil, as opposed to what occurs in other countires^3^,^7^,^8^,^10^, ^11^, ^12^, ^13^, ^14^, ^15^, ^16^, ^17^; these tests in this country are usually performed by speech therapists in clinical settings. Relative time was equivalent to testing infants under sedation, that is, keeping ideal condition from beginning to end. Pauses were needed to maintain this condition and respect the infants physiological state as testing in this study was done with infants in natural sleep. Absolute time was the sum of the relative time and the pauses. Thus, absolute time was the true duration of testing with the chosen method. Absolute and relative times differed significantly in tone burst ABR and ASSR testing. Knowing this difference is important to define the testing protocols when reviewing other methods and comparing data.

Except for the duration of the ASSR, there were no statistically significant differences between the preterm and term groups in the comparative analysis. The literature contains some differences in comparisons of similar infants, in which preterm infants have worse thresholds, slower responses, and longer latencies compared to term infants^20^,^22^. However, these studies evaluated preterm infants with a mean post-conception age of 35 weeks, whereas this age was 47 weeks in our study. It is probable that maturation from the 35^th^ to the 47^th^ weeks was responsible for similar findings in preterm and term infants in the present study, which differs from previous work^20^,^22^.

Further studies of different population groups with other methods to study ASSR and tone burst ABR will help understand these techniques in greater detail to expand their clinical applicability.

## CONCLUSION

The results of this study led to the conclusion that the two methods - tone burst ABR and ASSR - may be applied clinically at 2 kHz in infants; the mean test duration was 20 minutes. Preterm infants require more time for ASSR, but in general showed no differences compared to term infants.
